# Canine Parainfluenza Virus Infection in a Dog with Acute Respiratory Disease

**DOI:** 10.3390/vetsci9070346

**Published:** 2022-07-09

**Authors:** Marco Cordisco, Maria Stella Lucente, Alessio Sposato, Roberta Cardone, Francesco Pellegrini, Delia Franchini, Antonio Di Bello, Stefano Ciccarelli

**Affiliations:** 1 Department of Veterinary Medicine, University of Bari “Aldo Moro”, 70010 Valenzano, Italy; marco.cordisco@uniba.it (M.C.); mariastella.lucente@uniba.it (M.S.L.); roberta.cardone@uniba.it (R.C.); francesco.pellegrini@uniba.it (F.P.); delia.franchini@uniba.it (D.F.); stefano.ciccarelli@uniba.it (S.C.); 2 Istituto Zooprofilattico Sperimentale della Puglia e della Basilicata, 72028 Torre S. Susanna, Italy; alessio.sposato@izspb.it

**Keywords:** CPIV, CIRDC, kennel cough, respiratory syndrome, bronchoscopy, dry cough

## Abstract

**Simple Summary:**

A one-day history of dry paroxysmal cough, associated with retching, induced by canine parainfluenza virus without the simultaneous presence of other pathogens, has been reported in a vaccinated household dog. The dog did not show nasal discharge or fever, but it was possible to evoke a dry cough through the palpation of the trachea. Radiographic findings of the thorax showed a diffuse unstructured interstitial pattern with the involvement of multiple lung lobes. Trachea-bronchoscopy and broncho-alveolar lavage were carried out. Edema without exudate and congested mucosa from the larynx to bronchi were observed. Cytological evaluation was negative for the presence of inflammatory or infectious processes. Nucleic acids were extracted from the collected specimens; biomolecular investigations tested positive only for canine parainfluenza virus and negative for all other pathogens associated with “kennel cough”. At first, the afebrile onset and the coughing fits suggested the presence of a foreign body, a common occurrence in Southern Italy during summer. The clinical signs and the absence of findings by cytology have directed the clinicians towards the correct diagnosis, with the support of biomolecular assays, which are fundamental to avoid underestimating the circulation of this virus, even in owned dogs.

**Abstract:**

The canine infectious respiratory disease complex (CIRDC) is an endemic respiratory syndrome caused by different bacterial and viral pathogens. This report describes a case of canine parainfluenza virus infection in a vaccinated household dog with an acute respiratory symptom (dry cough), who underwent clinical and endoscopic investigations for a suspected foreign body. Cytological investigations carried out on the broncho-alveolar lavage fluid (BALF) tested negative for the presence of inflammatory or infectious processes and could have been misleading the clinicians. By the molecular analyses (PCR) carried out on the BALF, canine parainfluenza virus was exclusively detected without the simultaneous presence of other respiratory pathogens associated to CIRDC. This case report emphasizes the role of molecular diagnostics in the differential diagnosis of respiratory diseases, in order to avoid underestimating the circulation of the parainfluenza virus in the canine population.

## 1. Introduction

Canine infectious respiratory disease complex (CIRDC), also called “kennel cough”, is an endemic respiratory syndrome observed in densely housed environments, due to overpopulation and continuous introduction of pathogens [1]. As previously reported, the most common pathogens associated with CIRD development includes canine adenovirus 2 (CAV-2), canine distemper virus (CDV), canine herpesvirus (CHV), canine parainfluenza virus type 2 (CPIV) and *Bordetella bronchiseptica* (*Bb*). New pathogens have been implicated in the development of CIRDC, such as canine influenza virus (CIV), canine respiratory coronavirus (CRCoV) and canine pneumovirus (CnPnV).

CPIV, a member of the family Paramyxoviridae, is highly contagious, and therefore endemic worldwide. It was first isolated together with other pathogens from laboratory dogs with respiratory disease [2]. The virus is transmitted via aerosol and is responsible for a respiratory infection without systemic diffusion. The virus replicates in the nasal mucosa, the pharynx, the trachea and the bronchi, inducing moderate lesions and petechial hemorrhages in the lungs. Other infectious agents, particularly *Bb*, are generally associated with CPIV [3].

This case report describes clinical, radiographic, endoscopic and laboratory findings in a dog with an acute respiratory disease induced by CPIV, without the simultaneous presence of other pathogens.

## 2. Materials and Methods

### 2.1. Case Presentation and Sample Collection

An 8-year-old entire male, mixed breed dog, was presented with one-day history of dry persistent paroxysmal coughing episodes, associated with retching. The owners reported that the dog appeared tired and did not want to feed regularly. The dog was annually vaccinated with EURICAN^®^ DAPPi-Lmulti, a multivalent parenterally administered vaccine protecting against several core and non-core diseases. During the physical examination, the dog did not show nasal discharge or pyrexia; its respiratory rate was 36 acts/minute (apm) and its heart rate was 110 beats/minute (bpm) at 39.4 °C body temperature. A dry cough was evoked through the palpation of the trachea. Chest auscultation showed reinforced vesicular murmur in all lung fields. The dog was evaluated by a baseline assessment consisting of a complete blood count, serum biochemistry, which did not show any hematological or serum significant alterations. Radiographic findings in latero-lateral (LL left and right recumbency) were performed.

Subsequently, the dog underwent anesthesia to perform a trachea-bronchoscopy and broncho-alveolar lavage (BAL). The patient was preoxygenated for 5 min before anesthesia induction. A fibro-bronchoscope with a length of 85 cm and 5.2 mm diameter at the tip was used for BAL analysis, with an endoscopy video system (Tele Pack Vet × Led^®^ video system, equipped with Telecam^®^ camera and Karl Storz fibro-broncoscope). The additional technical characteristics of the fiberscope were as follows: total length of 113 cm, ventral dorsal angular deflection of 195°/105°, visual direction 0°, visual opening angle of 110°, and internal diameter of the working channel equal to 2.3 mm. During endoscopic examination, the dog was placed in sternal recumbency on a foam mattress.

After the endoscopic procedures, BAL was performed using sterile 0.9% NaCl saline solution (two boluses of 25 mL each per lobe) injected through the irrigation channel of the endoscope and immediately collected for analysis.

Cytological and microbiological analyses were performed on the sample collected; the specimen was cultured on Columbia blood agar (CBA), MacConkey agar (MCK) and mannitol salt agar (MSA) (Liofilchem, Roseto degli Abruzzi (TE), Italy) and incubated at 37 °C for 48 h in aerobic conditions.

BAL fluid (BALF) was collected in Falcon^®^ conical tubes and centrifuged at 400× *g* for 7 min. The pellet obtained from BALF centrifugation was recovered and smeared on slides. Slides were stained with May–Grunwald–Giemsa stain for cytological evaluation.

### 2.2. Sample Processing and Molecular Analyses

Both DNA and RNA nucleic acids were extracted from 200 μL of the BALF sample, using the IndiSpin^®^ Pathogen Kit (manufactured by QIAGEN for INDICAL, INDICAL BIOSCIENCE, product of Germany), according to the producer’s instructions.

The RNA extracts were tested by RT-PCR for the detection of canine parainfluenza virus (CPIV) [4]. A 667-bp fragment was amplified using primers PNP1 (5′-AGTTTGGGCAATTTTTCGTCC-3′) and PNP2 (5′-TGCAGGAGATATCTCGGGTTG-3′), which correspond to 120–140 and 786–766 nucleotide positions for the nucleocapsid protein gene sequence of paramyxovirus simian virus 5 (SV5) (GenBank accession no. AF052755), respectively.

Reverse transcription and the amplification reaction were carried out in a single step using SuperScript One-Step RT-PCR kit (Invitrogen, Life Technologies, Milan, Italy). The RT step included an incubation at 50 °C for 30 min, followed by denaturation of the enzyme at 94 °C for 2 min. The following amplification reaction included 40 cycles of denaturation at 94 °C for 1 min, annealing at 50 °C for 40 s and elongation at 72 °C for 1 min. The final extension step was carried out at 72 °C for 10 min.

Analysis of the PCR products was performed by electrophoresis in a 1.5% agarose gel containing a fluorescent nucleic acid marker (GelRed^®^ Nucleic Acid Gel Stain, Biotium). The gel was run at 120 V for 25 min and visualized under fluorescent light on the Gel Doc EZ imaging system (GelDocTMEZ System with Image Lab software, Bio-Rad Laboratories).

The RNA extracts obtained from BALF were also tested by real-time RT-PCR assays able to detect canine distemper virus (CDV) [5], canine respiratory coronavirus (CRCoV) [6], canine pneumovirus (CnPnV) [7] and canine influenza virus (CIV) [8].

The DNA extracts obtained from BALF were screened by PCR assay for *Bb* [9], *Streptococcus equi* subsp. *zooepidemicus* [10], *Mycoplasma cynos* and *Mycoplasma canis* [11,12], and by real-time PCR for canid herpesvirus 1 (CaHV-1) [13] and canine adenovirus 1 (CAdV-1) and 2 (CAdV-2) [14] (Table 1).

Five days later, the same investigations were repeated on the nasal swabs of the dog.

### 2.3. Sequencing

The CPIV 667-bp amplicon obtained using PNP1 (5′-AGTTTGGGCAATTTTTCGTCC-3′) and PNP2 (5′-TGCAGGAGATATCTCGGGTTG-3′), was sent to the PRIMM laboratories (Primm Eurofins) in order to perform SANGER sequencing.

The bioinformatics software platform Geneious Prime (Biomatters LTD, Auckland, New Zealand) and the online database tools BLAST [15] and FASTA [16] were used to analyze the sequences obtained. Genome sequences of the parainfluenza strains were retrieved from GenBank and aligned using the Clustal Omega tool from the European Molecular Biology Laboratory [17].

## 3. Results

**Figure 1:**
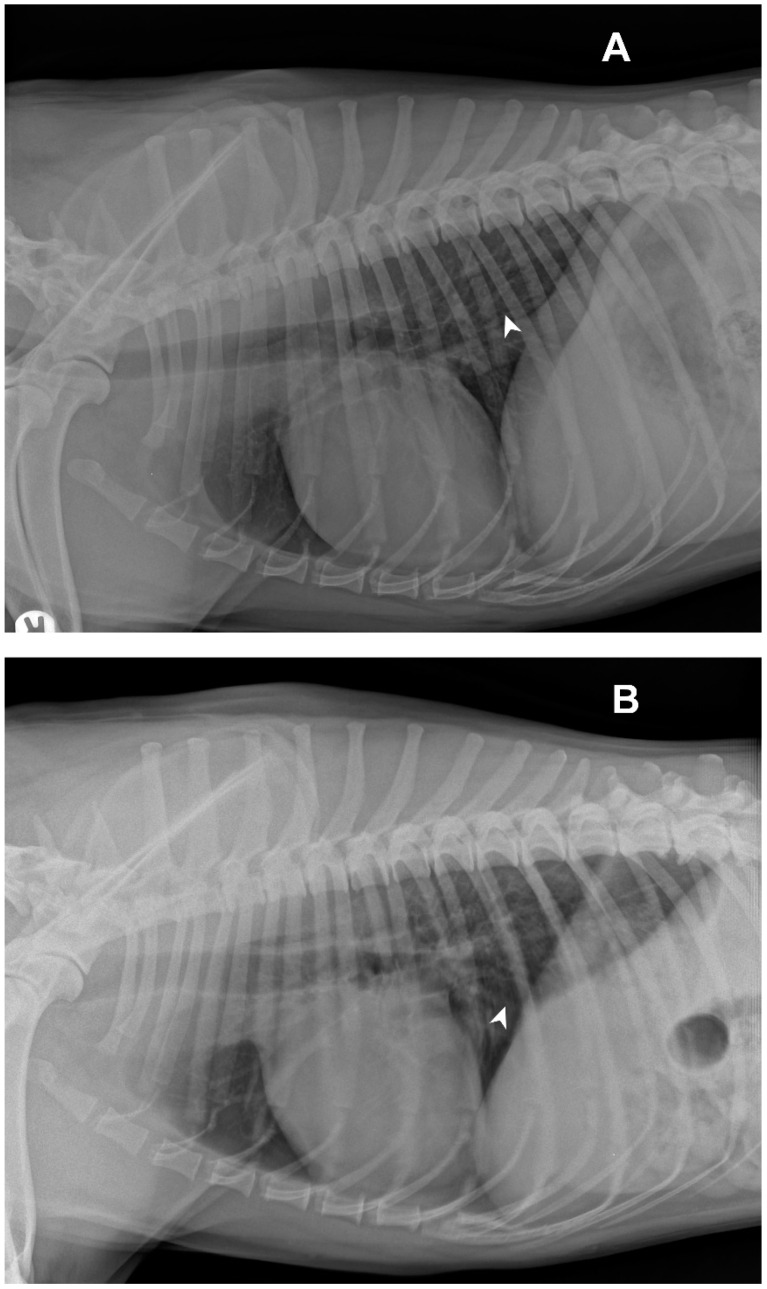
Right (**A**) and left (**B**) latero-lateral views of the dog: white arrows indicate examples of areas with unstructured diffuse interstitial pattern.

Radiological investigations of the thorax showed a diffuse unstructured interstitial pattern. The dog had a perihilar distribution and multiple lung lobes were affected (Figure 1).

**Figure 2:**
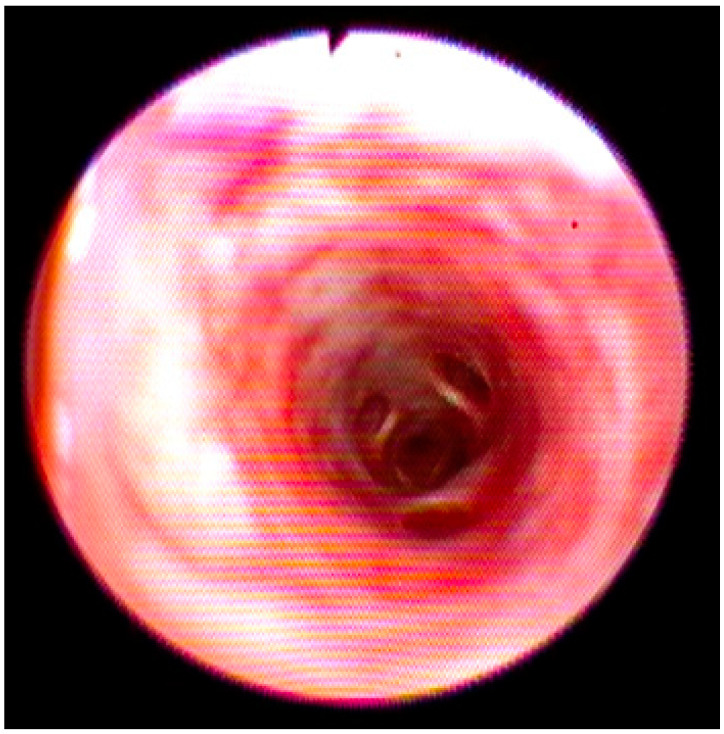
Endoscopic view of the principal broncho with congested mucosa without exudate.

During endoscopic examination, a diffuse edema and congested mucosa from the larynx to the trachea and bronchi were observed. Moreover, an increase in the vascular texture that was widespread, especially in the bronchi, without the presence of exudate was detected (Figure 2).

The microbiological analysis did not show significant growth, even after prolonged incubation.

**Figure 3:**
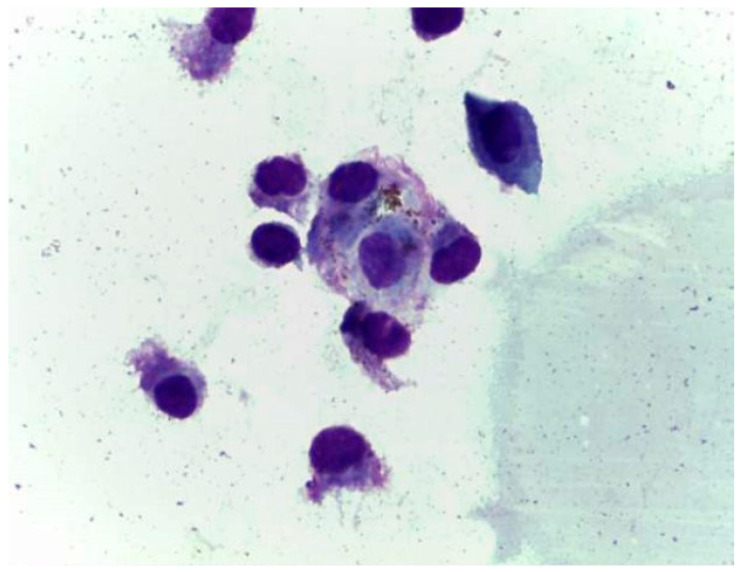
Normal cytological findings with presence of proteinaceous material, normal cylindrical ciliated cells, absence of etiological agents.

Cytological evaluation performed on the sample collected after BAL evidenced normal ciliated cells and rare macrophages (Figure 3).

Following the biomolecular investigations, the BALF sample tested positive by the specific RT-PCR assays for CPIV. Tests for CDV, CRCoV, CnPnV, CIV, CaHV-1, CAdV-1, CAdV-2, *S. equi* subsp. *zooepidemicus*, *M. cynos*, *M. canis*, *B. bronchiseptica* resulted negative.

The sequence analysis of the detected amplicon was successfully performed using BlastN; by comparison with the reference CPIV sequences available in the GenBank database, the amplicon sequence showed the highest nt identity (98.9%) with GenBank accession number AY581307, related to the CPIV clone T65 nucleocapsid protein (NP) gene sequence, a wild-type strain identified by Erles et al. in the United Kingdom in 2004 [4].

From a clinical point of view, the dog was treated with hydrocodone 0.25 mg/kg PO every 8 h (q8h) for 4 days, until the cough disappeared. Five days after the first sampling and one day after the pharmacological treatment’s conclusion, nasal swabs were lastly collected from the dog and tested negative for all the mentioned pathogens, including CPIV.

## 4. Discussion

Canine infectious respiratory disease is a widespread disease caused by single or multiple infectious agents involved sequentially or synergistically to cause mild to moderate or severe disease. The pathogens more frequently associated with CIRD include canine adenovirus type 2, canine parainfluenza virus, *Bordetella bronchiseptica* and canid herpesvirus 1. CIRD is typically responsible for outbreaks of disease in shelters, kennels and rehoming centers, in relation to the high density of dog populations, but can also occasionally occur in singly household dogs [18,19,20].

There is evidence that CPIV is still an important pathogen in CIRDC in Europe. In 2016, Decaro and co-authors investigated the presence of CPIV in dogs with acute CIRD, dogs exposed to CIRD and CIRD convalescent dogs, and reported detection prevalence of 20.5%, 4.5% and 2.6%, respectively [1], suggesting that CPIV is commonly found both in dogs with signs of CIRDC and in asymptomatic dogs [1,4,21]. Several studies confirm that CPIV is the primary etiological agent involved in canine respiratory diseases [22,23].

Transmissible respiratory diseases, including CPIV, are traditionally spread through infected aerosol and droplets during coughing and sneezing or by contact with fomites [19,20].

CPIV natural infection usually occurs within the epithelial cells of the upper respiratory tract and is generally self-limiting. Clinical signs, when present, generally occur 2–10 days after infection and include dry and paroxysmal harsh cough for 2–6 days, nasal discharge, retching, tonsillitis, pharyngitis, with or without pyrexia [20,24,25,26]. The frequent involvement of multiple etiological agents included in CIRD further increases the severity of the disease and makes it difficult to recognize symptoms attributable to CPIV alone [24]. The administration of cough suppressants, such as hydrocodone, can be considered a valid treatment for a nonproductive cough, but it should be avoided in animals with productive cough in order to not compromise the normal clearance of bacteria [20,26].

The viral shedding for CPIV may occur for 8–10 days after infection and molecular investigations should be carried out on respiratory secretions, nasopharyngeal swabs and bronchoalveolar lavage fluid [20,24,25]. However, vaccines against CPIV do not induce sterilizing immunity; subsequently, they seem to elicit an incomplete protection [19]. Nevertheless, both the severity of the disease and the viral shedding seem to be reduced in dogs with a history of vaccination [18,19,24].

In this case report, an acute and afebrile onset was observed, characterized by the presence of coughing fits that could at first suggest the presence of a foreign body, a common occurrence, especially in the summer season in southern Italy.

Diagnostic imaging highlighted the elements compatible with CPIV infection, as also reported in the literature [20,26]. The cytological investigations carried out on the BALF were negative for the presence of inflammatory or infectious processes and these data should direct the diagnosis towards other etiologies. By laboratory investigations, the dog tested positive only for CPIV; this result highlights the risk to underestimate the spread of CPIV, favored by the incomplete protection provided by vaccinations [18,19,21].

Biomolecular investigations carried out 5 days after the first sampling and the fact that the dog tested negative for CPIV confirms that the duration of virus shedding is limited to a few days after infection.

## 5. Conclusions

The results achieved through molecular biology assays represent a very important element to increase knowledge about the prevalence of CPIV, also in household dogs.

Clinical, endoscopic and cytological investigations, even when the tests are negative, should direct clinicians towards a correct differential diagnosis supported by molecular diagnostics.

Therefore, it is important to include canine parainfluenza virus in the diagnostic algorithm of canine respiratory diseases.

## Figures and Tables

**Table 1:** Summary of the oligonucleotide primers used in this study.

Virus and Primer	Sequence (5′ to 3′)	Product Size (bp)
Canine distemper virus ^a^		
CDV-F	AGCTAGTTTCATCTTAACTATCAAATT	83
CDV-R	TTAACTCTCCAGAAAACTCATGC	
Canine respiratory coronavirus ^b^		
BCoV-F	CTGGAAGTTGGTGGAGTT	85
BCoV-R	ATTATCGGCCTAACATACATC	
Canine pneumovirus ^c^		
CnPnV-For	AAGATAAATTCTTCTATGAAAACAGAATGA	110
CnPnV-Rev	CCCATCGTAAGTGAGGTTTCTATT	
Canid herpesvirus 1 ^d^		
CHV-For	ACAGAGTTGATTGATAGAAGAGGTATG	136
CHV-Rev	CTGGTGTATTAAACTTTGAAGGCTTTA	
Canine influenza virus ^e^		
M-Flu1	CTTCTAACCGAGGTCGAAACGTA	147
M-Flu2	GGATTGGTCTTGTCTTTAGCCA	
*Bordetella bronchiseptica* ^f^		
FLA 2	AGGCTCCCAAGAGAGAAAGGCTT	237
FLA 4	TGGCGCCTGCCCTATC	
*Streptococcus equi* subsp. *zooepidemicus*^g^		
sodA equi/zooep-F	CAGCATTCCTGCTGACATTCGTCAGG	235
sodA equi/zooep-R	CTGACCAGCCTTATTCACAACCAGCC	
Canine adenovirus ^h^		
CAV-F	AGTAATGGAAACCTAGGGG	80
CAV-R	TCTGTGTTTCTGTCTTGC	
*Mycoplasma cynos* ^i^		
Myc1-For	CACCGCCCGTCACACCA	227
M. cynos-Rev	GATACATAAACACAACATTATAATATTG	
*Mycoplasma canis* ^i^		
Myc1-For	CACCGCCCGTCACACCA	247
M. canis-Rev	CTGTCGGGGTTATCTCGAC	

^a^ Oligonucleotide positions that refer to the sequence of canine distemper virus strain Onderstepoort. ^b^ Oligonucleotide positions that refer to the sequence of BCoV strain Mebus (GenBank accession no. U00735). ^c^ Oligonucleotide positions that refer to the sequence of CnPnV strain dog/Ane4/USA/2008 (GenBank accession no. HQ734815). ^d^ Oligonucleotide positions that refer to the sequence of CHV-1 glycoprotein B (GenBank accession no. AF361073). ^e^ Primers and probe were designed based on the sequence homology of the matrix gene (segment 7) among different influenza A virus isolates, belonging to subtypes from H1–H13 isolated in avian, human, swine and equine hosts. ^f^ An upstream sequence of the flagellin gene (*fla* gene), including the sigma consensus site and putative ribosome-binding site, was used as a target DNA region. ^g^ Oligonucleotide positions that refer to the sequences of *S. equi* subsp. *zooepidemicus* sodA gene (GenBank accession no. Z95902) encoding superoxide dismutase A. ^h^ Oligonucleotide positions that refer to the sequences of CAdV-1 (GenBank accession no. AC000003) and CAdV-2 (GenBank accession no. AC000020) within the highly conserved hexon gene. ^i^ Regarding *Mycoplasma* spp., primers were designed based on the sequence of 16S/23S rRNA intergenic spacer (IGS) species-specific regions, which are as follows: *M. spumans*, AF538684; *M. opalescens*, AF443612; *Mycoplasma* sp. strain VJC 358, AY246564; *M. gateae*, AF443609; *M. cynos*, AF538682; *M. molare*, AF443611; *M. molare*, AF538683; *M. maculosum*, AF443610; *M. opalescens*, AF538961; *Mycoplasma* sp. strain HRC 689, AF527624; *Mycoplasma arginini*, AF443604; *M. cynos*, AF443606; *M. felis*, AF443608; *M. edwardii*, AF443607; *M. canis*, AF443605.

## Data Availability

Data are contained within the article.
